# Anti-Oral Pathogens of *Tecoma stans* (L.) and *Cassia javanica* (L.) Flower Volatile Oils in Comparison with Chlorhexidine in Accordance with Their Folk Medicinal Uses

**DOI:** 10.3390/medicina55060301

**Published:** 2019-06-24

**Authors:** Hamdoon A. Mohammed, Marwa M. Abdel-Aziz, Mostafa M. Hegazy

**Affiliations:** 1 Pharmacognosy Department, Faculty of Pharmacy, Al-Azhar University, Cairo 11371, Egypt; mostafahegazy@azhar.edu.eg; 2 Medicinal Chemistry and Pharmacognosy Department, College of Pharmacy, Qassim University, Buraydah 51452, Saudi Arabia; 3 Regional Centre for Mycology and Biotechnology (RCMB), Al-Azhar University, Cairo 11371, Egypt; marwaemam.17@azhar.edu.eg

**Keywords:** *Cassia javanica*, *Tecoma stans*, volatile oils, chlorhexidine, *Streptococcus mutans*, oral pathogens, antimicrobial activity

## Abstract

*Background and Objectives*: Teeth decay and plaque are complicated problems created by oral pathogens. *Tecoma stans* (L.) and *Cassia javanica* (L.) are two ornamental evergreen plants widely distributed in Egypt. These plants are traditionally used for oral hygienic purposes. This study aims to elucidate the volatile oil constituents obtained from the flowers of these plants and evaluate the antimicrobial activity of these volatile oils against specific oral pathogens in comparison to chlorhexidine. *Materials and Methods:* The flowers obtained from both plants were extracted by n-hexane. GC-MS spectrometry was used to identify the constituents. Minimum inhibitory concentrations (MICs) were measured using tetrazolium salt (2,3-bis[2-methyloxy-4-nitro-5-sulfophenyl]-2H-tetrazolium-5-carboxanilide) (XTT). *Results:* GC-MS analysis revealed the presence of 32 and 29 compounds, representing 100% of the volatile constituents of *Tecoma stans* and *Cassia javanica*, respectively. The GC-MS analysis showed more than 60% of the volatile oil constituents are represented in both plants with different proportions. Chlorhexidine exerted stronger activity than tested plants against all microorganisms. *Cassia javanica* flower extract was more active against all tested microorganisms than *Tecoma stans*. Of note was the effect on *Streptococcus mutans*, which was inhibited by 100% at 12.5 and 25 µg/mL of *Cassia javanica* and *Tecoma stans*, respectively. The growth of *Lactobacillus* *acidophilus* was also completely inhibited by 25 µg/mL of the *Cassia javanica* extract. MIC90 and MIC were also calculated, which revealed the superiority of *Cassia javanica* over *Tecoma stans* against all tested oral pathogens. *Conclusion:* *Cassia javanica* flower volatile oils showed a potential anti-oral pathogen activity at relatively low concentrations. Also, *Cassia javanica* and *Tecoma stans* demonstrated a strong activity against tooth decay’s notorious bacteria *Streptococcus mutans*. Both plants can be potential substituents to chlorhexidine. Formulating the constituents of these plants in toothpastes and mouthwashes as anti-oral pathogen preparations can be an interesting future plan.

## 1. Introduction

Microorganisms are considered a major cause of disease induction in the world, in which the disease arises when a specific microorganism fits to the site where it can grow, colonize, and release its toxins [1]. Oral pathogens play a major role in the formation of dental plaque and, subsequently, dental caries and general periodontal diseases [2]. Health problems related to oral pathogens may extend outside the oral cavity to reach the cardiovascular system [3]. Also, oral care reduces the currency of influenza [4], pneumonia, and the mortality ratio in elderly individuals [5]. Plaque growth is regularly initiated due to bacterial colonization and adherence to the solid substratum, forming a microbial biofilm [6]. Bacterial colonies have the ability to ferment carbohydrates to produce acids. The produced acids are responsible for demineralization and cavities initiation in teeth [7,8]. Natural products, particularly plant-derived products, have been used for prophylactic and/or treatment of dental caries and plaques. For instance, the toothbrush tree “*Salvadora persica*” or Arak is the most well-known tree used in this regard [9,10,11]. Also, numerous plant extracts and their constituents have been examined for their effects on dental caries by inhibiting bacterial growth [12,13,14,15,16]. Chlorhexidine is highly effective against a wide range of oral pathogens such as *Lactobacillus acidophilus* and *Streptococcus mutans*. The long-term usage of chlorhexidine is accompanied by several side effects, such as tanning the tooth with yellowish brown discoloration. In addition, irritation and/or inflammation may arise due to chlorhexidine mouthwashes, which may be exaggerated to damage of mouth mucosa [17,18].

*Cassia javanica* L. is an ornamental tree that belongs to the Fabaceae family and produces a mass of beautiful pink flowers, so it is commonly known as the pink shower [19]. The plant is traditionally used in the treatment of fever, cold, gastric pain, constipation, diabetes mellitus, malaria [20], and also used for oral hygiene to stop bad breath [21]. There are various phytochemical classes in the plant, such as anthraquinone glycosides [21], flavonoids [22], alkaloids, sterols, tannins, saponins [20], and volatile oil [23]. *Cassia javanica* has many pharmacological activities, such as antimicrobial [23], anti-herpes simplex virus type 2 (anti-HSV-2) [24], antioxidant, anti-inflammatory [25], and antidiabetic [26] activities. In addition, its honey has antifungal and anticancer activities [27].

*Tecoma stans* (L.) (Family: Bignoniaceae) is an ornamental tree with gorgeous yellow bell-shaped flowers. Its fast growth and propagation rates cause it to be regarded as an invasive tree like those in South Africa and Namibia [28]. It is used in traditional medicine as a remedy for diabetes mellitus, digestive problems, stomach pain, intestinal worms, and snake bite [29]. *Tecoma stans* contains alkaloids, flavonoids, tannins, terpenes, phytosterols [30], and irridoids [31]. Many biological activities were confirmed, especially those based on folk use as a hypoglycemic and hypolipidemic agent for diabetes mellitus [32]. In addition, *Tecoma stans* has wound-healing [33], antioxidant, antimicrobial [34], analgesic, anti-inflammatory [35], nephroprotective [36], and cardioprotective activities [29].

*Cassia javanica* (L.) and *Tecoma stans* (L.) Juss. are common trees in Egyptian gardens and are covered by numerous flowers in the blooming season. The availability of these trees and their enormous flower production gave the idea of finding an applicable biological activity. Accordingly, we aimed here to identify the flower volatile oil constituents obtained from these plants. In addition, the antimicrobial activity of the constituents of these plants against oral pathogens was evaluated, considering that these plants are used in folk medicine for oral hygiene.

## 2. Materials and Methods

### 2.1. Plant Collection and Method of Extraction

*Cassia javanica* (L.) and *Tecoma stans* (L.) Juss. flowers were collected in September 2018 from different gardens in Cairo, Egypt. The plants were kindly identified as *Cassia javanica* (L.) and *Tecoma stans* (L.) Juss. flowers by Dr. Mohamed El Gebali and plant engineer Therese Labib (Herbarium Section, Al-Orman Garden, Giza, Egypt) before shade drying in open air at room temperature. Accurately, 500 g of the grinded dried flowers from both plants were sonicated with n-hexane solvent using a sonicator for 24 h (ultrasonic, Branson 5800). The n-hexane extracts were vacuum dried under reduced pressure using a Rotavapor (BUCHI R-300) and the dried residues were stored in at −20 °C.

### 2.2. Gas Chromatography-Mass Spectroscopy (GC-MS) Analysis

The GC-MS analysis was performed on a Shimadzu GCMS-QP 2010 (Koyoto, Japan) equipped with an Rtx-5MS capillary column (30 m × 0.25 mm inner diameter (i.d). × 0.25 µm film thickness) (Restek, Bellefonte, PA, USA). The oven temperature was kept at 50 °C for 3 min (isothermal), programmed to 300 °C at 5 °C/min, and kept constant at 300 °C for 10 min (isothermal); the injector temperature was 280 °C. Helium was used as a carrier gas with a constant flow rate set at 1.37 mL/min. Diluted samples (1% v/v) were injected with a split ratio of 15:1 and the injected volume was 1 μL. The MS operating parameters were as follows: interface temperature, 280 °C; ion source temperature, 220 °C; EI mode, 70 eV; scan range, 35–500 amu. For compound identification, components were identified based on their retention indices and by matching their mass spectra with the National Institute of Standards and Technology (NIST-11), the Wiley library database, as well as published data in the literature (Adams 2004). Retention indices (RI) were calculated relative to a homologous series of n-alkanes (C8-C40) injected under the same conditions.

### 2.3. Identifications of the Cassia javanica and Tecoma stans Constituents

The flower constituents extracted by n-hexane from both plants were identified based on the experimental retention index (RI) calculated in comparison to a series of n-alkenes (C8 to C40) and retention indices obtained from the literature under similar GC experimental conditions. The identifications of the compounds were carried based on retention time, mass fragmentation pattern, and according to mass spectral libraries (NIST-11 and Wiley library database). Relative percentages of the constituents were calculated from the area under the peaks obtained from GC chromatograms.

### 2.4. Microorganisms

Oral pathogens; *Streptococcus mutans* ATCC 35668, *Porphyromonas gingivalis* ATCC 33277, *Lactobacillus acidophilus* ATCC 4356, and *Candida albicans* ATCC90028 obtained from the American Type Culture Collection were used in the antimicrobial assay.

### 2.5. Determination of the Minimal Inhibitory Concentration (MIC) Using XTT Assay

The MICs were determined using the micro-dilution method described by Tunney et al., 2004 [37]. The microbial inoculates were prepared, and the suspensions were adjusted to 106 CFU/mL. The n-hexane extracts and the standard drug (chlorhexidine) were prepared in dimethyl sulfoxide (DMSO). Subsequently, twofold dilutions were performed in a 96 well plate. Each well of the microplate included 40 μL of the growth medium brain heart infusion (BHI), 10 μL of inoculum, and 50 μL of the diluted extracts and standard compounds. DMSO was used as a negative control. The plates were incubated at 37 °C for 24 h. After that, 40 μL of tetrazolium salt (2,3-bis[2-methyloxy-4-nitro-5-sulfophenyl]-2H-tetrazolium-5-carboxanilide) (XTT) was added to the wells. The plates were incubated in the dark place for 1 h at 37 °C, after which colorimetric change in the XTT reduction assay was measured using a microtiter plate reader (Tecan Sunrise absorbance reader; Tecan UK, Reading, United Kingdom) at 492 nm. The percentage of inhibition was calculated using the following equation:

Inhibition %=(1−Absorbance oftest/Absorbance of control) × 100

Standard error means were calculated from three independent assays.

## 3. Results

The flower part of *Cassia javanica* and *Tecoma stans* was extracted by n-hexane to measure the antimicrobial effect of these plants against common oral pathogens in accordance with their traditional uses [38,39].

The constituents of n-hexane extracts obtained from both plants were investigated by GC and GC-MS spectrometry. The compounds identities were based on the retention index (RI), retention time (*RT*), and mass fragments obtained from the GC-MS chromatogram (GC chromatograms are shown in the Supplementary Materials), which were compared to the literature data as well as data from NIST-11 and the Wiley library database. In accordance with the aforementioned data, the results obtained from the GC-MS analysis ensured that the flower part of these plants contains 20 similar compounds. However, these compounds were represented in these plants by different concentrations (Table 1). For instance, decane and 2-methyldecane were represented by 6.06 and 2.57% in *Tecoma stans* and by 0.89 and 1.10% in *Cassia javanica*, respectively. Furthermore, some of the constituents were only represented in one of the plants while they were absent in the other. For example, p-cymen-8-ol was represented only in *Tecoma stans* with 23.73% while α-terpineol and 1,2-benzenedicarboxylic acid, mono(2-ethylhexyl) ester were represented in *Cassia javanica* with 29.32 and 6.71%, respectively. Table 1 shows the results for the volatile oil analysis of *Tecoma stans* and *Cassia javanica*, which indicate that monoterpene hydrocarbons and monoterpene alcohols represent the majority of the volatile constituents in these plants.

**Figure 1:**
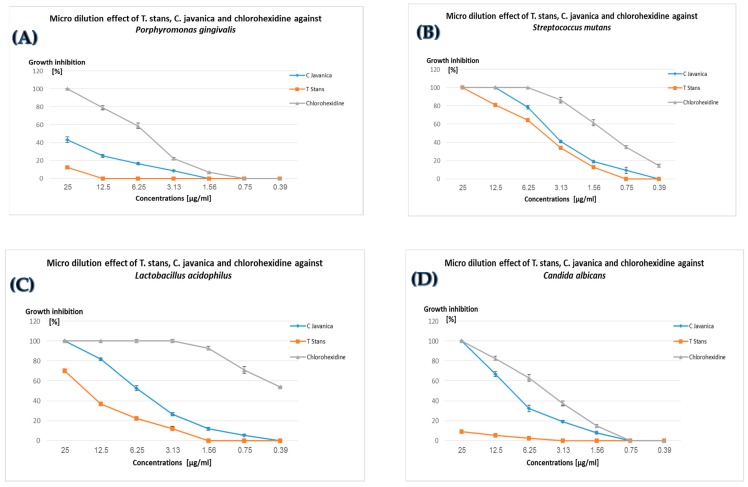
Percentage of microbial growth inhibition at different concentrations of *Cassia javanica*, *Tecoma stans*, and chlorhexidine against *Porphyromonas gingivalis* (**A**), *Streptococcus mutans* (**B**), *Lactobacillus acidophilus* (**C**), and *Candida albicans* (**D**). The results are expressed as the mean ± standard deviation (SD) obtained from three independent experiments.

The antimicrobial assay was conducted by the rapid determination method of tetrazolium salt (2,3-bis[2-methyloxy-4-nitro-5-sulfophenyl]-2H-tetrazolium-5-carboxanilide) (XTT) color reduction percentage, which is inversely proportional to the bacterial cell viability [37]. The results shown in Figure 1A–D for the n-hexane extracts of *Cassia javanica* and *Tecoma stans* in addition to those of the standard oral disinfectant “chlorhexidine” [50] against oral microbial pathogens indicate that the n-hexane extract of *Cassia javanica* was more active against all pathogens compared to the extract of *Tecoma stans*. For instance, *Cassia javanica* at a concentration of 3.13 µg/mL inhibit the growth of *Porphyromonas gingivalis*, *Lactobacillus acidophilus*, and *Candida ablicans* by 8.75, 26.47, and 19.16%, respectively, as compared to 0, 12, and 0% inhibition by *Tecoma stans* extract at the same concentration. The results in Figure 1 also reveal that the standard chlorhexidine was 2 to 10 times more active than n-hexane extracts from both plants, which were exaggerated—especially in the case of *Porphyromonas gingivalis*, which was inhibited by 12.7, 43.25, and 100% at 25 µg/mL of *Tecoma stans*, *Cassia javanica*, and chlorhexidine, respectively. In addition, the standard chlorhexidine inhibited the growth of *Lactobacillus acidophilus* at 0.39 µg/mL by 54% compared to 0% inhibition by *Tecoma stans* and *Cassia javanica* at the same concentration.

The anti-oral pathogenic effect of n-hexane extracts from *Cassia javanica* and *Tecoma stans* in addition to the positive control, chlorhexidine, was displayed in the form of MIC and MIC90. These represent the minimal concentration required to kill 100 and 90% of the microorganism, respectively (Table 2). The results summarized in Table 2 ensure the superiority of *Cassia javanica* over *Tecoma stans* n-hexane extract in the activity against tested oral pathogens. The MIC and MIC90 values were not recorded for *Tecoma stans* against the growth of *Porphyromonas gingivalis* and *Candida albicans* at the maximal experimental concentration (100 µg/mL).

## 4. Discussion

Natural products, particularly plant-derived secondary metabolites, have contributed to health promotions and enhanced humans’ life quality. These secondary metabolites include essential oils, different types of glycosides, alkaloids, and so forth. Regarding essential oils, they are famous antimicrobial agents against a broad spectrum of bacterial and fungal strains [51,52].

Exploring nature to find an effective treatment for oral health with minimal or no side effects caused by synthetic compounds is a great challenge. Chlorhexidine has many local side effects, the most common one being brown discoloration of the teeth, some restorative materials, the oral mucosa, and notably the tongue dorsum, which is an intraoral cosmetic problem [17,18,53].

The present work represents the volatile constituents of *Cassia javanica* and *Tecoma stans* extracted by n-hexane solvent. *Cassia javanica* and *Tecoma stans* are traditionally used for oral hygienic purposes due to their activity as antimicrobial agents [38,39]. The spectrometric analysis of *Cassia javanica* and *Tecoma stans* n-hexane extracts revealed that more than 60% of the volatile oil constituents of these plants are similar. Specifically, the identities of 20 compounds were quite similar out of the 29 and 32 identified compounds in *Cassia javanica* and *Tecoma stans*, respectively (Table 1). Meanwhile, large differences were recorded in the proportions of these compounds in the plants, as apparent in Table 1 for the proportions of decane, 3-carene, squalene, and stigmastan-3,5-diene.

The determination of antimicrobial activity revealed that the standard oral disinfectant chlorhexidine was more active against all oral pathogens than the n-hexane extract of tested plants. Also, the antimicrobial assay ensured that *Cassia javanica* was more active than *Tecoma stans* against all tested oral pathogens, which may be related to the presence of the monoterpene alcohols α-terpineol and 1,2-benzenedicarboxylic acid, mono(2-ethylhexyl) ester in relatively high concentrations in *Cassia javanica*. 1,2-Benzenedicarboxylic acid, mono(2-ethylhexyl) ester, which was identified only in *Cassia javanica* (6.71%), was reported in a similar concentration (6.55%) in *Muscodor tigerii*, which inhibits the growth of a broad spectrum of bacterial and fungal microorganisms [54]. Furthermore, α-terpineol, which was reported for its strong activity as an antimicrobial agent against oral pathogens and used as a component in toothpastes [55], was identified as a major compound in *Cassia javanica* (29.32%) while it was absent in *Tecoma stans*. Furthermore, *Cassia javanica* n-hexane extract at 12.5 µg/mL inhibits the growth of *Streptococcus mutans*, which plays a major role in dental decay, by 100% [56]. Also, a 100% inhibition in the growth of *Candida albicans* and *Lactobacillus acidophilus* was achieved by 25 µg/mL of *Cassia javanica* n-hexane extract. The potentiated activity for *Tecoma stans* was observed against *Streptococcus mutans* (100% inhibition) and *Lactobacillus acidophilus* (70% inhibition) at a concentration of 25µg/mL. This potential antimicrobial activity of *Tecoma stans* may be attributed to the high proportion of monoterpene alcohols (30.37%), particularly p-cymen-8-ol, which represents 23.73% of the total *Tecoma stans* volatile constituents. It is worth mentioning that p-cymen-8-ol is represented in the anti-oral pathogenic plant *Cyperus articulates* [57].

## 5. Conclusions

The phytochemical constituents of n-hexane extracts obtained from flowers of *Cassia javanica* and *Tecoma stans* indicate major similarities in identity of these plant volatile oils, including more than 60% of the constituents. Meanwhile, major differences in the proportion of these constituents were recorded. The antimicrobial record in this study ensured that *Cassia javanica* flower n-hexane extract might play a major role in the antimicrobial activity of this plant against oral pathogens, as also indicated by its use in folk medicine for oral hygiene. *Tecoma stans* flower extract exerted some activity, especially against tooth decay’s notorious bacteria *Streptococcus mutans*. Although *Cassia javanica* and *Tecoma stans* were less effective against oral pathogens than chlorhexidine, these plants might be safer for human use because they are naturally derived sources. Accordingly, these plants can be potential substitutes for chlorhexidine. Our future plan is to formulate the constituents of these plants in toothpastes and mouthwashes for anti-oral pathogens.

## Figures and Tables

**Table 1:** Chemical constituents of the n-hexane extracts of *Cassia javanica* and *Tecoma stans* flowers.

No	Compound Name	*RT*	*RI* ^exp^	*RI* ^rep^	^%R^ *Tecoma stans*	^%R^ *Cassia javanica*
1	Decane	9.161	989	891	6.06	0.89
2	3-Carene	9.870	1012	1012	1.21	0.36
3	m-Cymene	10.160	1021	1021	0.62	-
4	(Z)-β-Ocimene	10.890	1044	1043	0.93	-
5	Heptyl acetate	10.980	1047	1047	0.92	-
6	(Z)-β-Ocimene	11.075	1050	1050	0.99	0.35
7	2-Methyldecane	11.190	1053	1051	2.57	1.10
8	γ-Terpinene	11.395	1060	1059	0.96	0.45
9	2,9-Dimethyldecane	12.350	1090	1086	15.87	11.46
10	Nonanal	12.875	1106	1107	1.62	-
11	Myrcenol	13.180	1116	1116	1.98	-
12	Fenchol	13.205	1117	1117	-	0.98
13	*trans*-Pinene hydrate	13.420	1124	1123	1.33	0.80
14	neo-Isopulegol	14.025	1143	1143	1.37	-
15	*trans*-Verbenol	14.070	1144	1144	1.41	1.73
16	Z-Tagetone	14.190	1148	1147	1.64	1.33
17	Citronellal	14.325	1152	1153	5.09	3.79
18	β-Pinene oxide	14.525	1159	1158	2.26	1.28
19	Artemisyl acetate	15.035	1175	1174	-	0.90
20	Terpinen-4-ol	15.176	1179	1177	-	0.50
21	p-Cymen-8-ol	15.455	1188	1188	23.73	-
22	α-Terpineol	15.490	1189	1189	-	29.32
23	cis-Dihydrocarvone	15.845	1200	1198	3.60	3.11
24	Pulegone	16.600	1226	1227	0.75	-
25	Citronellol	16.634	1228	1228	-	0.59
26	Cumin aldehyde	16.985	1240	1239	0.69	-
27	Neral	17.045	1242	1242	0.67	-
28	Carvone	17.180	1247	1248	0.88	0.55
29	Piperitone	17.320	1252	1253	2.77	2.42
30	Methyl citronellate	17.510	1258	1258	1.59	1.39
31	Linalyl acetate	17.580	1261	1261	2.95	2.58
32	Geranial	17.795	1268	1268	-	0.27
33	Limonen-10-ol	18.375	1288	1289	0.55	0.60
34	Palmitic acid	34.465	1951	1953	1.32	-
35	4-Hexadecyl hexanoate	42.185	2367	2362		0.57
36	Pentacosane	44.860	2533	2521	1.78	
37	1,2-Benzenedicarboxylic acid, mono(2-ethylhexyl) ester	44.895	2535	2521	-	6.71
38	Heptacosane	46.980	2669	2666	6.44	6.80
39	Octacosane	48.450	2764	2738	-	1.34
40	Squalene	49.850	2854	2831	4.76	10.51
41	Stigmastan-3,5-diene	52.560	3028	3040	0.67	7.33
Total percentage					100	100
Monoterpene hydrocarbons					29.12	14.61
Monoterpene alcohols					30.37	34.02
Esters					5.46	12.15
Aldehydes					8.07	4.06
Ketones					9.64	7.41
Long chain hydrocarbons					13.65	25.98

^Exp^ Experimental retention index (*RI*) using a series of n-alkanes (C10–C40) calculated under identical experimental conditions. ^Rep^ Reported retention index according to the National Institute of Standards and Technology (NIST) library and published literature data and calculated under identical experimental conditions as the references [40,41,42,43,44,45,46,47,48,49]. ^%R^ The relative percentage of individual volatile components according to the peak area and calculated from the GC (Gas chromatography) chromatogram; *RT* = retention time.

**Table 2:** The minimal inhibitory concentrations required to kill 50, 90, and 100% of the oral pathogens from *Cassia javanica*, *Tecoma stans*, and chlorhexidine.

Pathogen	*Cassia javanica*			*Tecoma stans*			Chlorhexidine		
	MIC50	MIC90	MIC	MIC50	MIC90	MIC	MIC50	MIC90	MIC
*Porphyromonas gingivalis*	30.13 ± 1.48	79.03 ± 0.78	100	96.50 ± 2.2	nd	nd	5.51 ± 0.17	16.25 ± 0.57	25
*Streptococcus mutans*	3.89 ± 0.07	9.57 ± 0.21	12.5	4.77 ± 0.02	18.49 ± 0.39	25	0.31 ± 0.01	0.99 ± 0.11	1.56
*Lactobacillus acidophilus*	5.93 ± 0.22	21.2 ± 0.23	25	17.39 ± 0.33	47.6 ± 1.8	100	0.36 ± 0.01	1.5 ± 0.03	3.13
*Candida albicans*	9.43 ± 0.41	17.5 ± 0.23	25	nd	nd	nd	1.16 ± 0.03	4.47 ± 0.18	6.25

nd = MIC and MIC90 were not detected until 100 µg/mL of the extract was employed; Minimum inhibitory concentration (MIC).

## Supplementary Materials

The following are available online at https://www.mdpi.com/1010-660X/55/6/301/s1, Figure S1: Gas chromatography chromatogram of *Tecoma stans* flowers volatile oils, Figure S2: Gas chromatography chromatogram of *Cassia javanica* flowers volatile oils.
