# Supplementary material for: Anti-Oral Pathogens of Tecoma stans (L.) and Cassia javanica (L.) Flower Volatile Oils in Comparison with Chlorhexidine in Accordance with Their Folk Medicinal Uses
Source: Medicina (Kaunas). 2019 Jun 24;55(6):301. doi: 10.3390/medicina55060301 (PMC6631167; doi:10.3390/medicina55060301)
Supplement: Supplementary file 1 [file medicina-55-00301-s001.pdf]

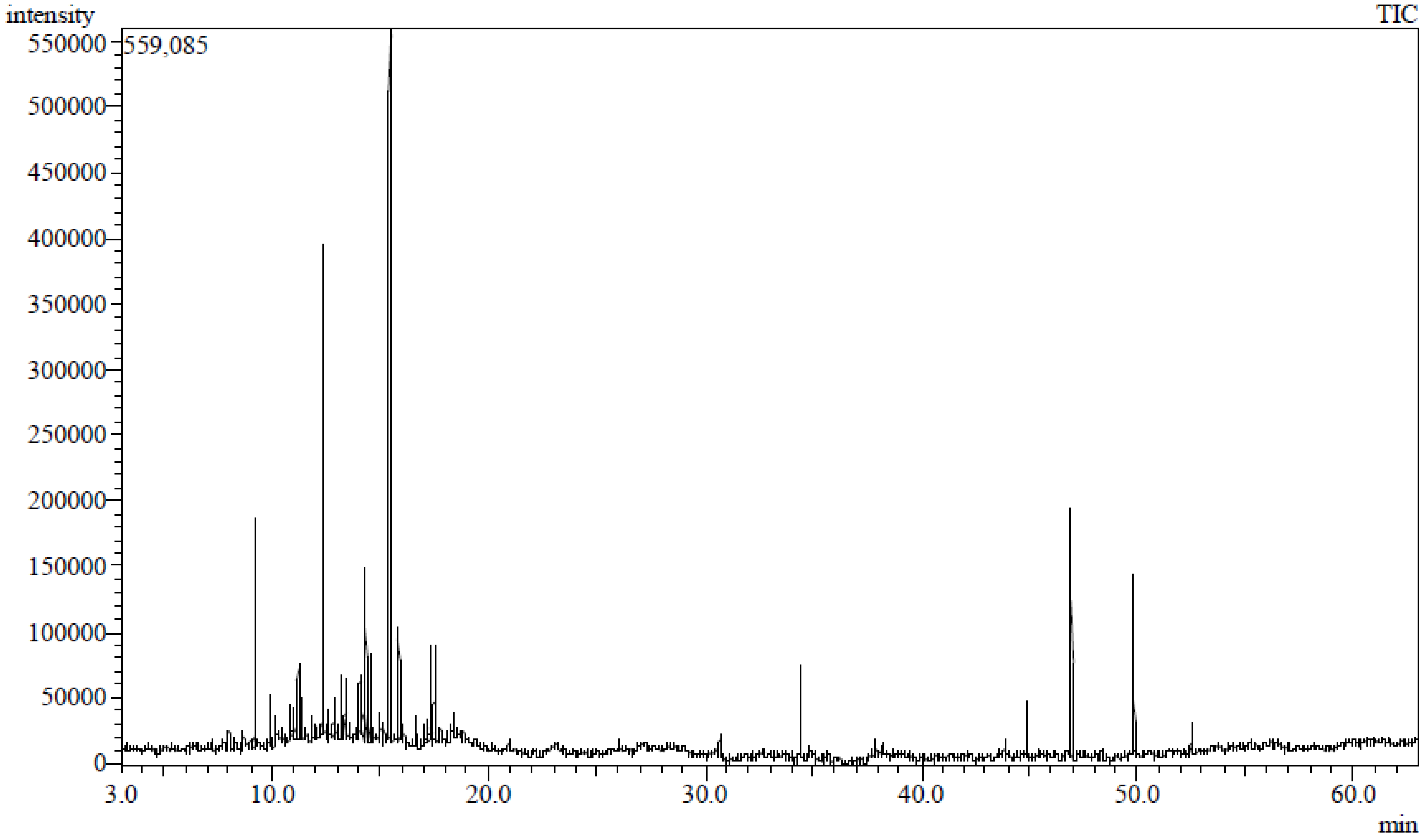

Figure 1 Gas chromatography chromatogram of *Tecoma stans* flowers volatile oils

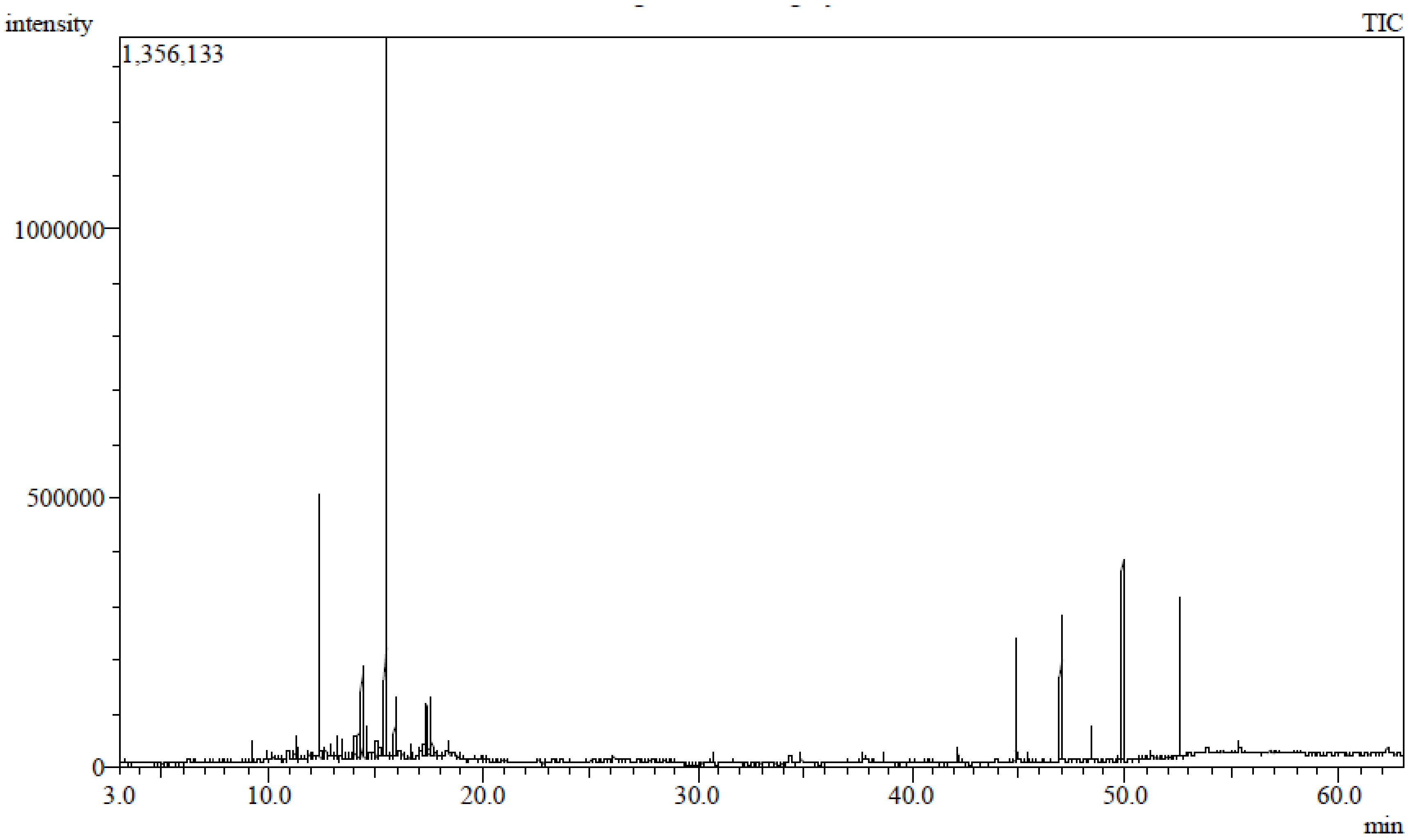

Figure 2: Gas chromatography chromatogram of *Cassia javanica* flowers volatile oils
